# Performance-based ability emotional intelligence benefits working memory capacity during performance on hot tasks

**DOI:** 10.1038/s41598-017-12000-7

**Published:** 2017-09-15

**Authors:** María José Gutiérrez-Cobo, Rosario Cabello, Pablo Fernández-Berrocal

**Affiliations:** 1 Department of Basic Psychology, Faculty of Psychology, University of Málaga, Málaga, Spain; 2 Department of Developmental and Educational Psychology, University of Granada, Granada, Spain

## Abstract

Emotional intelligence (EI) is the ability to perceive, use, understand, and regulate emotions. Higher scores on this ability measured through performance tests (but no through self-reports) appears to be related to better performance on “hot” (emotionally laden) cognitive tasks. However, there are relatively few studies concerning how EI may benefit the working memory capacity (WMC). Thus, the objective of this study is to analyse the relationship between EI (as measured through a performance-based ability test, a self-report mixed test, and a self-report ability test) and the WMC during the performance of hot and “cool” (i.e., non-emotionally laden) “2-back” tasks. 203 participants completed three EI tests as well as two 2-back tasks. The results provide evidence for better performance of higher EI participants (specifically in the managing branch) measured through the performance-based ability test, but only on the hot task. For the self-report mixed model, incongruent results were found, and no correlations were obtained using the self-report ability model. The implications of these findings are discussed in terms of the validity of the various EI models.

## Introduction

Emotional intelligence (EI) following the most relevant scientific model^1^ (p. 10) is defined as ‘… the ability to perceive accurately, appraise, and express emotion; the ability to access and/or generate feelings when they facilitate thought; the ability to understand emotion and emotional knowledge; and the ability to regulate emotions to promote emotional and intellectual growth’.

Since the EI construct was initially defined, many studies have been conducted in order to shed light on both its theoretical and practical aspects. This growing interest in the field of EI has given rise to numerous methods of evaluation, which, although not always covering the same aspect, have all been used in the study of EI. In an attempt to organize this EI literature, Joseph & Newman^2^ proposed three models of EI which can be distinguished by virtue of the type of instrument employed for assessing the construct: the *performance-based ability model*, the *self-report ability model* and the *self-report mixed model*.

The *performance-based ability model* understands EI as a combination of emotional aptitudes and is viewed as a form of intelligence. The method for assessing this model involves individuals resolving emotional problems through a performance test where there are correct and incorrect responses. The most representative instrument for evaluating this model is the “Mayer-Salovey-Caruso Emotional Intelligence Test” (MSCEIT)^3^ designed to measure the four branches of the ability EI model: perceiving, facilitating, understanding, and managing emotions. The *self-report ability model* also views EI as a form of intelligence as well as a group of emotional aptitudes. However, this model evaluates EI through self-report instruments where there are no correct and incorrect responses, but instead focuses on the subjective belief of the individual. A well-known instrument used within this perspective is the “Trait Meta-Mood Scale” (TMMS)^4^. Finally, the self-report mixed model views EI as a wider construct that includes motivations, personality factors, social skills and empathy. This model also employs self-report instruments such as the “Bar-On Emotional Quotient Inventory” (EQi)^5^.

One aspect that still remains unclear in the EI literature is the cognitive process underlying the construct. One particular question is whether individuals high in EI show better performance in tasks involving cognitive processes. In a recent attempt to answer this question, Gutiérrez-Cobo, Cabello and Fernández-Berrocal^6^ carried out a systematic review. These authors divided the results according to two variables: the EI model employed in the study (performance-based ability model, self-report ability model and the self-report mixed model) and the nature of the stimuli used in the cognitive task (hot or emotionally laden and cool or non-emotionally laden). They found that through the performance-based ability model (but not through self-reports) higher EI favoured performance on hot cognitive tasks. No such results were found with cool cognitive tasks, irrespective of the EI model employed. However, Gutiérrez-Cobo *et al*.^6^ pointed out two main limitations of their study. The first of these limitations involves the large variability of EI instruments employed; and whilst these instruments can be included in one of the three models, they show different psychometric properties and cover different numbers of variables. The second limitation is that given the lack of studies, all the cognitive processes (attention, decision making, memory, etc.) have been analysed together, and it is possible that each may be influenced by EI in a different manner, thus confounding the results and possible conclusions.

In order to address the limitation revealed in the work of Gutiérrez-Cobo *et al*.^6^, Gutiérrez-Cobo, Cabello and Fernández-Berrocal^7^ evaluated how each of the three EI models were related to a specific cool and hot cognitive process known as cognitive control ability. In particular, they employed the most representative instrument for each of the three EI models and a cool and hot Go/no-go task for measuring cognitive control. They found that higher scores on the managing branch of the performance-based ability model instrument of EI were correlated with a higher level of cognitive control ability in the hot task. In contrast, the use of self-reports yielded inconsistent results. Finally, no relationships were found between the cool Go/no-go task and any of the EI instruments. Thus, the results of Gutiérrez-Cobo *et al*.^7^ were consistent with those found in the previous literature^6^.

The next logical step could be to analyse other cognitive process, and one particular candidate of interest is the working memory capacity (WMC). The WMC allows us to temporarily store relevant information while inhibiting the processing of irrelevant stimulation^8^. Individual differences in WMC have been related to differences in cognitive functioning^9–11^. In addition, WMC has been studied in the clinical population. Thus, reduced WMC has been found in individuals with posttraumatic stress disorder^12^, with bipolar disorder^13^, with depressive^14^ or anxious symptoms^15,16^ in comparison with the non-clinical population.

The WMC has been traditionally measured through various tasks involving a processing and storage dual task where participants are required to recall certain information. For instance, in the Reading Spam test^17^, participants have to read a set of sentences and remember the last word of each sentence. The spam test usually varies from two to six sentences. Another task that is becoming increasingly popular for assessing WMC is the n-back task^18^. In a typical n-back task, participants have to decide whether a current stimulus had been presented *n* trials before. Thus, in a 1-back level, participants have to compare the current stimulus with the previous stimulus, whilst, for instance, in a 2-back level, the target stimulus has to be compared with the one shown two positions before. Unlike the Reading Spam test where participants are required to recall information, individuals in the n-back are involved in a recognition task. The n-back task is widely used in current studies.

There are some indications that EI could be related to the WMC, given the overlapping of brain regions involved in both processes. Thus, various studies have demonstrated how EI^19,20^ and the WMC^21,22^ are related to the prefrontal cortex (PFC). More recently, it has been commonplace to analyse how the benefits of the emotional working memory training (WMT) could produce transfer effects in emotion regulation ability, which is a core branch of EI. These studies support the transfer effect between emotional WMT and emotion regulation ability found when using behavioural and brain measurements^23–26^. For instance, Schweizer *et al*.^24^ show how WMT with an emotional (but not neutral) n-back task improved performance on a Stroop task. Engen and Kanske^25^ found that participants were able to reduce their emotional reactions to aversive film clips using cognitive reappraisal.

The present study has two main objectives. Firstly, we wanted to explore which of the three models of EI is more related to the hot WMC. Secondly, we wanted to analyse whether the relationship between EI and WMC is dependent on the emotional content of the task by evaluating the relationship between the three EI models and the cool WMC. Additionally, we were interested in evaluating if both the hot and cool 2-back tasks measured the same cognitive processes, as well as examining their differences in difficulty. To this end, we measured EI through three instruments representing each of the EI models, whilst WMC was measured using two 2-back tasks: a hot or emotionally laden and a cool or non-emotionally laden 2-back task. On the basis of the results of Gutiérrez-Cobo *et al*.^6^, Gutiérrez-Cobo *et al*.^7^ and the literature on WMT, we proposed three hypotheses. Firstly, we anticipated that higher scores on the performance-based ability model (but not on the self-report models) would be related to better performance on the hot 2-back task. Secondly, we expected to find no relationship between EI and the cool 2-back task, irrespective of the model employed. Finally, we predicted that both the cool and hot 2-back tasks will be correlated and that the hot cognitive task would have a greater level of difficulty than the cool task.

## Results

### Descriptive statistics and correlations among EI instruments

Descriptive analyses were conducted for the three EI instruments (Supplementary Table S1). In order to analyse how the three EI measures are related, correlational analyses were conducted (Supplementary Table S2). For the MSCEIT, the management scale correlated positively with the intrapersonal EQi-s scale and the clarity and repair TMMS scale. For the EQi-s, the interpersonal and adaptability scales correlated positively with the attention, clarity and repair TMMS scales. The stress management scale correlated negatively with the attention scale and positively with the clarity scale, and, finally, the intrapersonal scale correlated positively with the clarity and repair scales of the TMMS.

### Hot N-back Tasks and EI

#### Correlations

Correlational analyses were carried out between the three EI instruments and all the indices of the three conditions of the hot 2-back task in order to test our first hypothesis.

*MSCEIT:* We found significant and negative correlations between the managing branch of the MSCEIT and the MR index on the happy (r = −0.18, p = 0.02) and angry (r = −0.17, p = 0.03) conditions. For the RT and the accuracy indexes, we did not find any significant correlation. No significant correlations were found for the neutral condition.

*TMMS:* No significant correlations were found between the EI instrument and any of the three indices of the hot 2-back task.

*EQi-S:* We found a negative and significant correlation between the adaptability scale and the MR index on the neutral condition (*r* = −0.19, *p* < 0.02) and a near significant positive correlation between the accuracy index and the same scale (*r* = 0.15, *p* = 0.05). We did not find any correlation with the RT index and the EI instrument or with the angry and happy conditions.

#### Multiple regressions

In order to clarify the relations found for the three EI instruments in the MR and accuracy indices of the hot 2-back tasks, we carried out multiple regressions analyses.

For the angry condition, we found that the managing branch of the MSCEIT significantly predicted the MR scores. This predictor explained 2% of the variance (adjusted R^2^ = 0.02, F(1,166) = 5.08, p = 0.03). Specifically, higher scores on the managing branch significantly predicted less MR (β = −0.17, p = 0.03). No other EI scores predicted the MR index of this hot condition and no significant predictors were found for the accuracy index.

For the happy condition, we again found the managing branch of the MSCEIT to be the unique predictor of the MR score. This predictor explained 2% of the variance (adjusted R^2^ = 0.02, F(1,166) = 4.96, p = 0.03). Specifically, higher scores on the managing branch significantly predicted less MR (β = −0.17, p = 0.03). No other EI scores predicted the MR index of this hot condition and no significant predictors were found for the accuracy index of the happy condition.

Finally, for the neutral 2-back task, we found the adaptability scale of the EQi-S to be the unique predictor of the MR index of the neutral face condition. This predictor explained 3% of the variance (adjusted R^2^ = 0.03, F(1,166) = 5.85, p = 0.02). It was found that participants with higher scores in the adaptability scale showed less MR (β = −0.18, p = 0.02). The other EI index scores were not predictors of the MR index of the neutral condition. In addition, the accuracy index was not predicted by EI.

### Cool N-back Task and EI

#### Correlations

Correlational analyses were carried out between the three EI instruments and the cool 2-back task in order to test our second hypothesis.

*MSCEIT:* We did not find any correlation between EI and any of the indices of the cool 2-back task.

*TMMS:* No significant correlations were found.

*EQi-S:* Negative and significant correlations were found between the intrapersonal scale and the accuracy index (*r* = −0.16, *p* = 0.04). No other correlations were found.

#### Multiple regressions

In order to clarify the relations found for the three EI instruments in the MR and accuracy indices of the cool 2-back tasks, we carried out multiple regressions analyses.

The results for the accuracy index of the consonant condition of the cool task indicated that the intrapersonal scale of the EQi-S was the only predictor. This predictor explained 2% of the variance (adjusted R^2^ = 0.02, F(1,166) = 4.76, p = 0.03). It was found that participants with higher scores on the intrapersonal scale showed lower accuracy on the cool 2-bak task (β = −0.17, p = 0.03). The other EI index scores showed no significant association with the accuracy index of the cool task and no significant results were achieved for the MR index and any of the EI scales.

### Hot and cool 2-back Tasks

#### Correlations

First, in order to confirm that both hot and cool tasks measured the same cognitive processes (third hypothesis), we correlated the RT, MR and accuracy for each condition of both tasks (happy, angry, neutral, and consonants). We found that all the parameters of each condition were correlated, except the MR between the happy and the consonant condition (*r* = 0.01, *p* = 0.19). For the RT index, we found positive correlations between the angry and the happy (*r* = 0.77, *p* < 0.01), neutral (*r* = 0.82, *p* < 0.01) and consonant (*r* = 0.63, *p* < 0.01) condition; between the happy and neutral (*r* = 0.77, *p* < 0.01) and consonants (*r* = 0.61, *p* < 0.01) conditions, and between the neutral and consonant (*r* = 0.73, *p* < 0.01) conditions. For the accuracy index, we again found a positive correlation between the angry and the happy (*r* = 0.37, *p* < 0.01), neutral (*r* = 0.59, *p* < 0.01) and consonant (*r* = 0.31, *p* < 0.01) condition; between the happy and the neutral (*r* = 0.41, *p* < 0.01) and consonant (*r* = 0.22, *p* < 0.01) condition, and, between the neutral and consonant (*r* = 0.33, *p* < 0.01) condition. Finally, for the MR indices, we found a positive correlation between the angry condition and the happy (*r* = 0.37, *p* < 0.01), neutral (*r* = 0.50, *p* < 0.01) and the consonant (*r* = 0.23, *p* < 0.01) condition; between the happy and the neutral (*r* = 0.41, *p* < 0.01) condition, and, between the neutral and consonant (*r* = 0.27, *p* < 0.01) condition.

#### ANOVA

**Figure 1:**
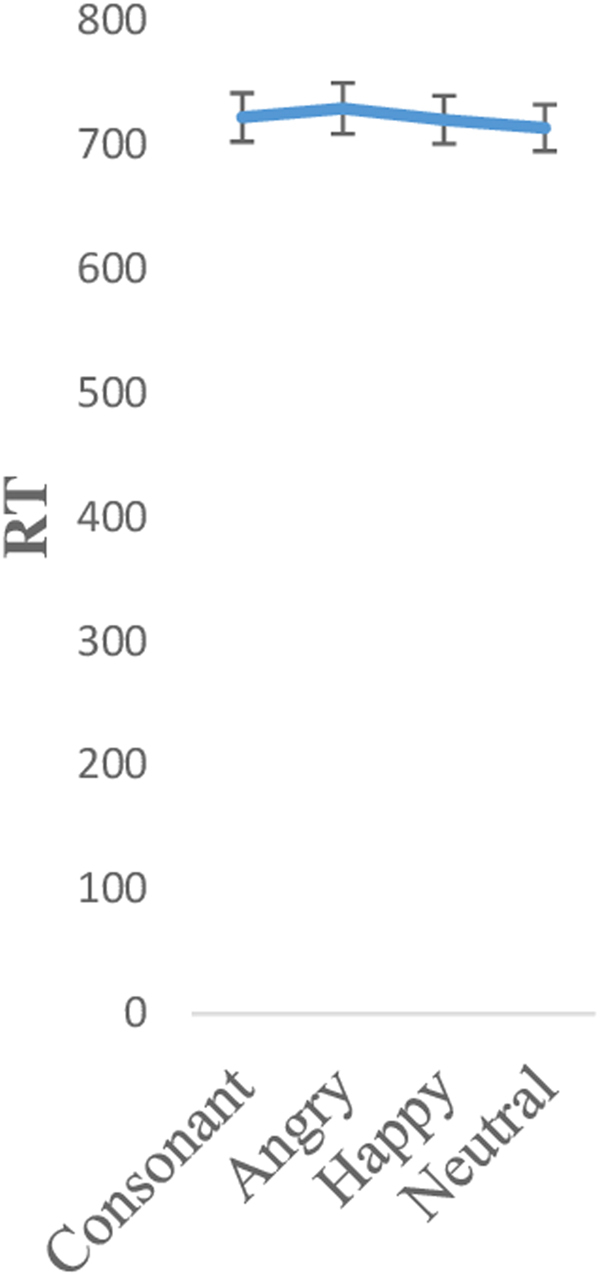
RT 2-back task per condition.

Secondly, in order to analyse differences in difficulty between conditions (third hypothesis), we carried out a within-subject ANOVA using the factor of condition (angry, happy, neutral and consonants) for each of the three parameters: RT, accuracy and MR. For the RT index (Fig. 1), we found that participants were faster in responding to the neutral (mean [SD]: 713.12 [18.65]), followed by the happy (mean [SD]: 719.78 [19.26]), then the consonant (mean [SD]: 721.61 [19.55]) and, finally, the angry (mean [SD]: 728.86 [20.43]) condition. However, these differences were not significant (F (3, 528) = 0.39, p = 0.73, η^2^_p_ < 0.01, Greenhouse-Geisser).

**Figure 2:**
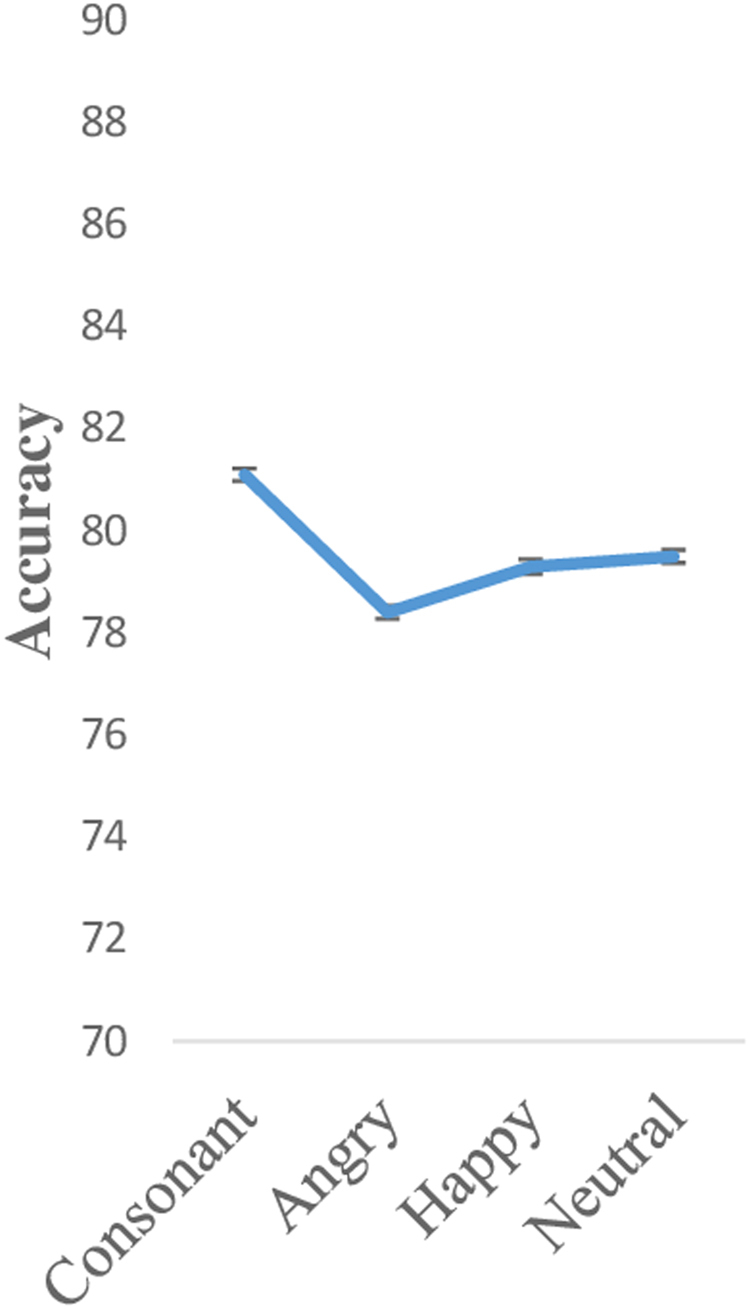
Accuracy 2-back task per condition.

For the accuracy index (Fig. 2), participants were more accurate in the consonant condition (mean [SD]: 81.1 [1.2]) than in the neutral condition (mean [SD]: 79.5 [1.3]), followed by the happy (mean [SD]: 79.3 [1.5]) and the angry condition (mean [SD]: 78.4 [1.3]). Again, these differences were not significant (F (3, 528) = 1.14, p = 0.33, η^2^_p_ = 0.01, Greenhouse-Geisser).

**Figure 3:**
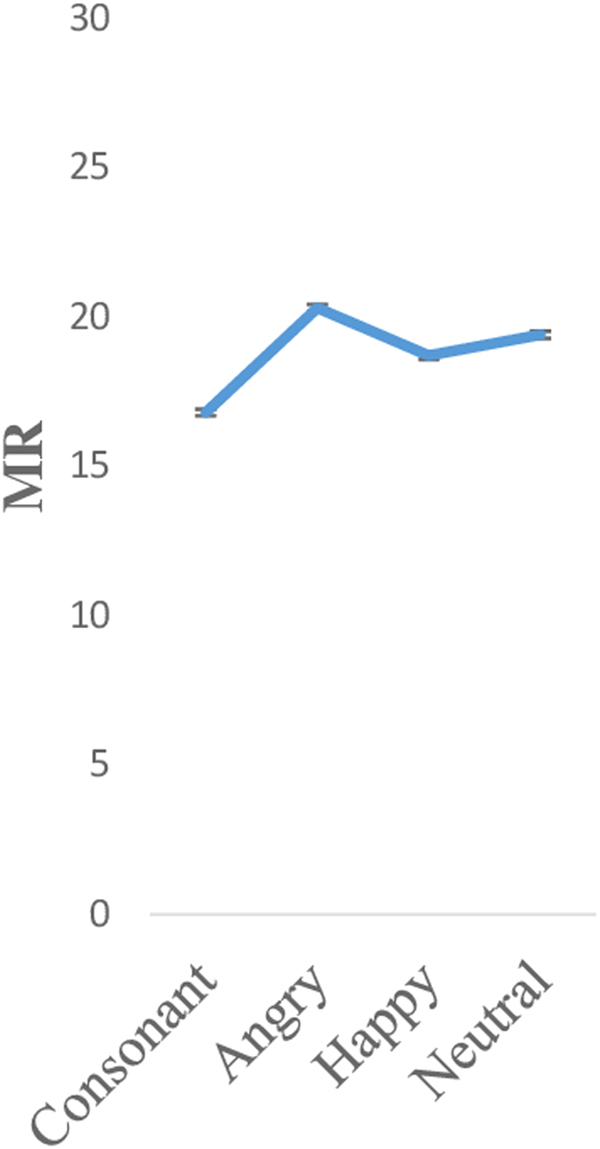
MR 2-back task per condition.

Finally, for the MR index (Fig. 3), participants achieved less MR in the consonant condition (mean [SD]: 16.8 [1.1]) than in the happy (mean [SD]: 18.7 [1.2]), followed by the neutral (mean [SD]: 19.4 [1.2]) and, finally, the higher MR was obtained in the angry condition (mean [SD]: 20.3 [1.2]). The main effect of MR approached significance (F (3, 528) = 2.33, p = 0.08, η^2^_p_ = 0.01, Greenhouse-Geisser). Post hoc t-tests showed that this main effect was due to differences between the angry and consonant condition (*t* = −2.49, *p* < 0.05).

## Discussion

The present experimental study aimed, firstly, to analyse the relationship between EI — measured through the three main models — and the hot WMC. Secondly, we were interested in analysing whether the relationship with the WMC was dependent on the emotional content of the task. In addition, we evaluated if both the hot and cool 2-back tasks measured the same cognitive processes, whilst examining the differences in level of difficulty between these tasks. For this purpose, we employed two 2-back tasks: a cool or non-emotionally laden task and a hot or emotionally laden task.

First, if we focus on the hot 2-back task results, we found that, consistent with our first hypothesis, when using the MSCEIT the results indicate that higher emotional management skills are related to lower MR in the angry and happy working memory task, which was the only predictive scale. These results are consistent with those of Gutiérrez-Cobo *et al*.^7^, where the managing branch was correlated with better cognitive control ability. This is also congruent with the previous literature regarding the transferable benefits of the WMT onto the ability to regulate emotions^23–26^. It is also interesting to note that the significant results were obtained with the angry and happy stimuli, and not with the neutral faces. In Gutiérrez-Cobo *et al*.^7^, the results were also primarily obtained when a happy and angry face was involved in the trial. Whilst these emotions (happiness and anger) are opposite in valence, they both share a high level of intensity. Thus, EI appears to be related to the processing of positive and negative high intensity stimuli. These results are unsurprising, given that in comparison with neutral emotions, these positive and negative emotions are better able to produce activation in the relevant brain regions^22^.

For its part, the higher scores in the adaptability scale of the EQi-S were related with better performance in the neutral face condition through lower MR. These results could suggest that the WMC for less intense stimuli may be related to the perceived ability to cope with the environment. However, these results are also inconsistent with the results of both Gutiérrez-Cobo *et al*.^6^ and Gutiérrez-Cobo *et al*.^7^. Finally, the TMMS showed no relationship with any indices of the hot 2-back task, which is again consistent with our first hypothesis.

With respect to the cool 2-back task, EI, when measured through the MSCEIT, was not related to any indices of the working memory task, thus suggesting that the benefit for higher EI participants is dependent on the emotional content of the task^6,7^ which is consistent with our second hypothesis. Again, the TMMS was not related to the cool task and, surprisingly, the intrapersonal scale of the EQi-S appeared to be counterproductive in the performance of this task.

The results with the MSCEIT are consistent with the conceptualization of the EI model proposed by Mayer, Caruso & Salovey^27^. These authors include EI as a member of the broader class of intelligence that focuses on hot information processing. This implies that EI could be a form of intelligence that is involved in the reasoning of significant information for the individual, which falls within the category of emotion. Thus, finding significant results on the emotional conditions in the 2-back task, but not in the neutral conditions, is compatible with the EI conceptualization. The fact that significant results are found only with the managing branch is also congruent with the idea that WMC may be linked to the ability to regulate our emotions^28^. WMC helps individuals to have a wider amount of information available, which then enables them to learn more about their experiences. In addition, WMC allows individuals to deal with greater resources for facing situations and controlling unwanted responses^29^.

In addition, our results showed how the total score of the MSCEIT was not related to the other two self-report instruments, whilst small correlations were found between the managing branch and the clarity and repair TMMS scale and the EQi:S intrapersonal scale (all *r* < 0.18). In contrast, stronger correlations were found between the two self-report EI instruments (from 0.22 to 0.59) which is consistent with the results of previous studies^7,30,31^ suggesting that, although the three models are labelled under the same term, the performance-based ability model does not cover the same construct as the self-report models.

Finally, when comparing the different conditions of the 2-back tasks, contrary to what was expected on the basis of our third hypothesis, we found no significant differences between the conditions. These results indicate that there were no differences in difficulty between the cool and hot tasks. Therefore, the lack of a significant correlation between MSCEIT and the cool 2-back task cannot be attributed to the ease with which participants could carry out the cognitive task.

A limitation of the present study is the lack of causal results. We cannot offer a causal effect of the results, given the cross-sectional nature of our study. Thus, in the future, it would be interesting to analyse how the EI training could improve the WMC and to then explore whether the effect of transfer of the WMT to the ability to regulate emotions is bidirectional. In addition, neurophysiological and neuroimaging measures need to be used in the future to stablish these causal results. Another important issue of future interest would be to investigate this issue in the clinical population. Previous findings have shown that WMC is impaired in depressed and anxious patients^14,15^. Although in healthy participants WMT helps individuals to reduce anger, fatigue or depression^32^, the evidence does not support the notion of beneficial transfer of the WMT to anxiety or depressive symptoms in the clinical population^33^. It could be interesting to evaluate whether EI training could improve the WMC as well as the specific symptomatology of various mental diseases.

A further two limitations are related to the sample. Firstly, the majority of the participants were females, and thus the results may have been affected by sex differences in EI^34^. Secondly, the sample was comprised of undergraduate students, who have specific characteristics and are supposed to have high intelligence levels, and are therefore not representative of the general population.

In conclusion, our study takes a step forward in the conceptualization of the EI construct within the domain of cognitive processes. More specifically, our findings show that, at least when using hot stimuli, the performance-based ability model is a better determinant measure for WMC than the self-report models. Consistent with previous literature, higher scores on the management branch of the MSCEIT are related to better performance on the emotional task but not on the cool task, suggesting the importance of emotional content. No significant results were found for the TMMS and, finally, inconsistent outcomes were obtained through the EQi-S. Future research should focus on the causality of the results, as well as on the relevant brain processes involved.

## Methods

### Participants and Procedure

A total of 203 (28% men) undergraduate psychologists from the University of Málaga (Spain), ranging in age from 19 to 48 years (Mean [SD]: 21.68 [3.27]) took part in the study. Due to the cut-off employed, the final sample was composed of 177 participants. The participants took part in the experiment in exchange for course credits. The study was carried out in accordance with the Declaration of Helsinki and ethical guidelines of the American Psychological Association, and all participants provided written informed consent. The study protocol was approved as part of the projects SEJ-07325 and PSI2012-37490 by the Research Ethics Committee of the University of Málaga. The study was developed in three different stages. Firstly, participants completed the performance-based ability model instrument. Secondly, they completed the self-report ability and mixed model instruments (6 and 8 participants did not complete these tests, respectively), and in a third stage they performed the cool and hot cognitive task. The cognitive tasks were carried out in a quiet room with 10 semi-isolated computers. The two cognitive tasks were created using the software E-prime 2.0.

### EI Instruments

*Mayer-Salovey-Caruso Emotional Intelligence Test* (MSCEIT v. 2.0)^3,35^. We employed the Spanish version of the MSCEIT as a performance-based ability test, which has similar psychometric properties to the English-version instrument^36^. This test has been validated for adults aged 17 and older. The MSCEIT consists of 141 items that measure the four branches of the Mayer and Salovey model^1^ previously described: *perceiving*, *facilitating*, *understanding*, and *managing* emotions. Perceiving emotions refers to the ability to perceive emotions in oneself and others as well as in other stimuli such as objects or art. Facilitating emotions is the ability to generate, use and feel emotions as necessary to communicate feelings or employ them in other cognitive processes. Understanding emotions refers to the ability to understand emotional information, how emotions combine and progress through relationship transitions, and to appreciate such emotional meanings. Finally, managing emotions is the ability to modulate them in oneself and others to promote personal understanding and growth. This instrument provides separate scores for each branch as well as a total EI score. These scores can be calculated based on expert or consensus norms and both correlate strongly with each other (r > 0.90)^37^. The test publishers have standardized the scores of the test (M = 100, SD = 15) and they have found that the reliability of the two halves is 0.93, based on the consensus criterion; while the test-retest reliability of the global MSCEIT is 0.86 after three weeks^38^. In the current study, we used the four scores of each branch as well as the total EI score, based on the consensus norms. Internal consistency ranged from 0.72 to 0.82 (perceiving = 0.78, facilitating = 0.72, understanding = 0.76, managing = 0.78, and total EI = 0.82). For these consistencies, we employed Cronbach’s alpha in the case of the total EI scores and the two-halves consistency in the case of the remaining scores.

*Trait Meta-Mood Scale* (TMMS)^4,39^. We employed the Spanish translation of the TMMS as a self-report ability test. This test has been validated in a university student population. The TMMS is a 24-item test that measures individuals’ beliefs in the attention, clarity, and repair dimensions^40^. Attention refers to the extent individuals tend to observe and think about their feelings and moods. Clarity indicates how people believe that they can understand one’s emotional states and, finally, repair refers to the individual’s beliefs about ability to regulate their emotional states. In our study, we found a Cronbach’s alpha of 0.90 for attention and clarity and 0.85 for repair, showing good reliability.

*Emotional Quotient Inventory–Short Form* (EQi:S)^41,42^. We employed the Spanish version of the EQi:S as a self-report mixed test. This test has been validated in a university student population. The EQi:S consists of 28 items that measure four dimensions of traits and self-concepts. These dimensions are intrapersonal, interpersonal, adaptability, and stress management. The intrapersonal scale refers to abilities such as emotional self-awareness, assertiveness or self-concept. The interpersonal dimension is focused on empathy, social responsibility and the management of interpersonal relationships. The adaptability scale refers to abilities of problem solving and flexibility, and, finally, the stress management scale is related to the stress tolerance and impulse control. This instrument provides separate scores for each dimension with reliabilities that ranged from moderate to high. In the present study, for the interpersonal scale, we found an acceptable level of reliability (Cronbach’s alpha = 0.70) while for adaptability (Cronbach’s alpha = 0.82), stress management (Cronbach’s alpha = 0.87), and intrapersonal scales (Cronbach’s alpha = 0.81) the reliability was strong.

### 2-Back tasks

**Figure 4:**
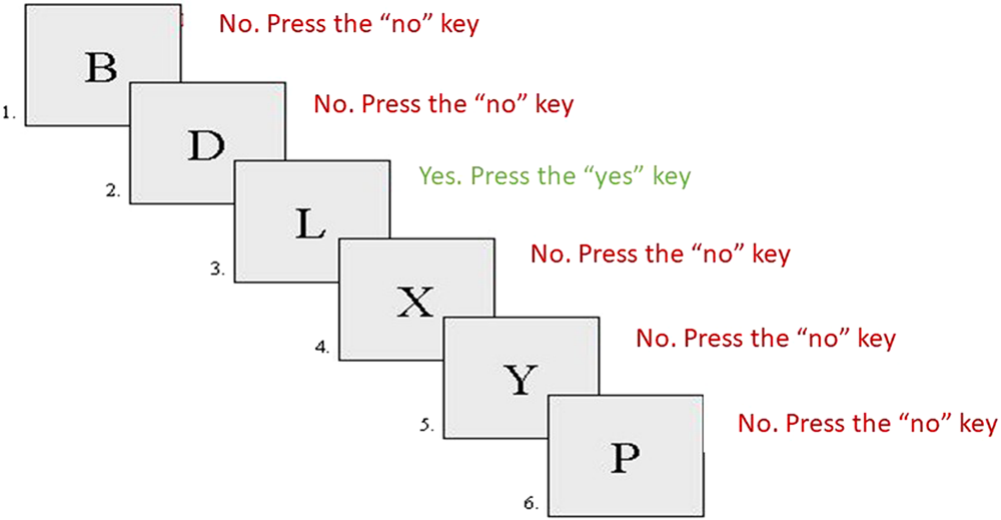
Cool 2-back task.

*Cool 2-back task*. This task was taken from Robinson & Fuller^43^. We employed only one of the levels of the task i.e. the 2-back level. The set of stimuli was composed of the following consonants: B, C, D, F, G, H, J, K, L, M, N, P, Q, R, S, T, V, W, Y, Z. In this task (Fig. 4), participants had to compare the current consonant with that presented two trials previously. If both consonants were identical, they had to press the “yes” key of the keyboard. Conversely, if the consonants were different they had to press the “no” key. There was one block with 30 trials and none of the three first trials could be a target (so the correct response was always “no”). Each of the trials began with the presentation of a consonant for 500 ms followed by a white screen of 3000 ms. Participants were allowed to give their responses until the white screen disappeared. All participants carried out a practice phase to familiarize themselves with the task before it began.

**Figure 5:**
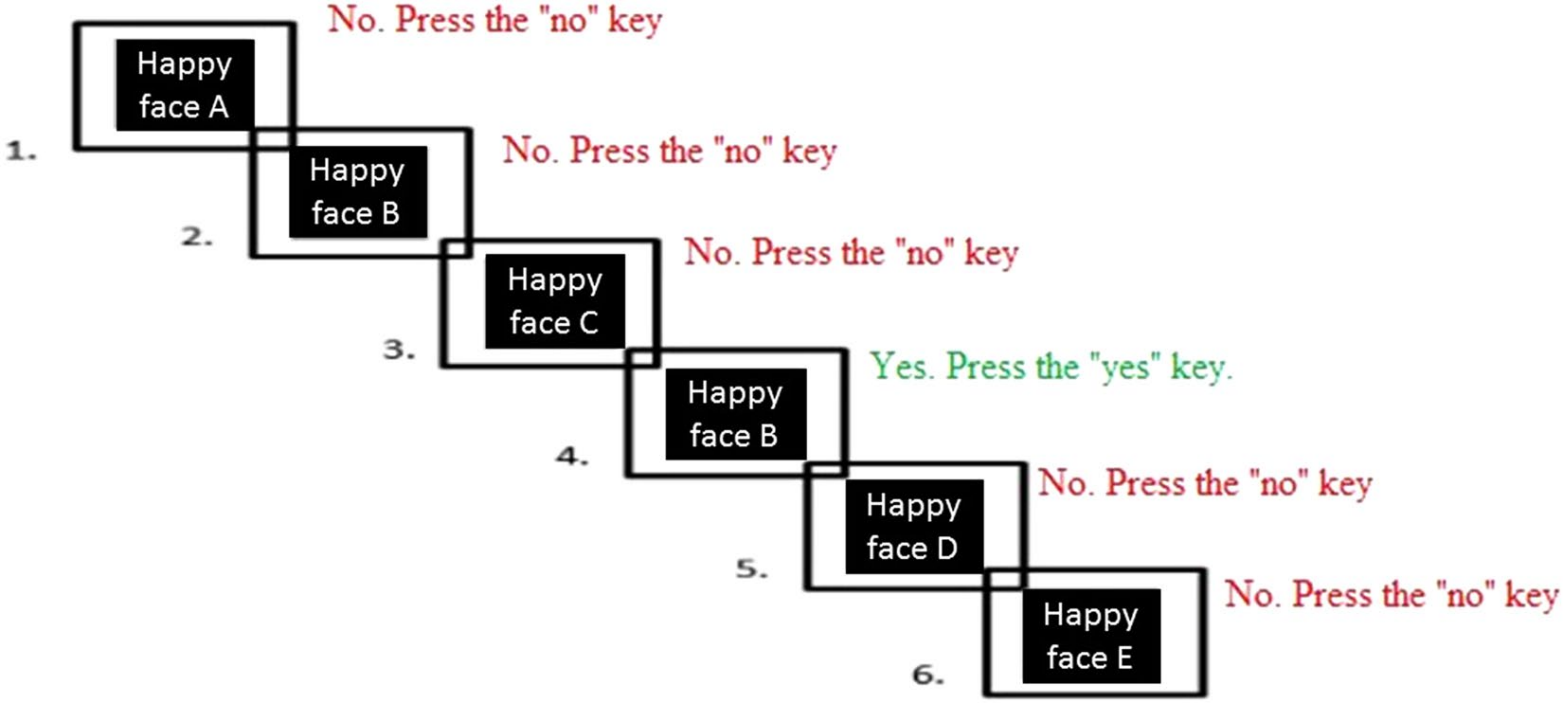
Hot 2-back task: Example of a happy condition.

*Hot 2*-*back task*. This task was an adaptation of the cool N-back task^43^. Again, we used the 2-back level (Fig. 5). However, unlike the cool task, the set of stimuli was composed of grey scale images of adult females and males with three different expressions: angry, happy, and neutral^44^. In this task, there were three blocks, categorized by the emotional expressions of the stimuli, with 30 trials in each block. Thus, one block was composed of happy faces, one of neutral faces and the other of angry faces. Again, participants carried out a practice phase to familiarize themselves with the task before it started.

### Data Plan Analyses

All analyses were conducted using SPSS 22.0 (IBM, USA). Participants with a total MSCEIT score below 85 were removed from the analyses. Preliminary descriptive and correlational analyses were conducted in order to compute the EI scores and the relations between them. To analyse the cool and hot 2-back tasks, we calculated reaction time (RT) for target trials, miss rate (MR) and accuracy indices. MRs were the number of “no” responses to the target stimulus, while accuracy was calculated as the number of “yes” responses to the target stimulus. Those participants presenting more than 2 SDs above the mean of total MR were also removed. RT was calculated only for correct trials and trials with no more tan +/− 3 SDs from the mean of each participant. Correlational analyses and multiple regressions were conducted in order to compare the three models of EI and hot and cool WMC. Finally, correlational and ANOVA analyses were carried out to compare the two working memory tasks.

## Electronic supplementary material

Supplementary information
