# Supplementary material for: Performance-based ability emotional intelligence benefits working memory capacity during performance on hot tasks
Source: Sci Rep. 2017 Sep 15;7:11700. doi: 10.1038/s41598-017-12000-7 (PMC5600979; doi:10.1038/s41598-017-12000-7)
Supplement: Supplementary file 1 — Supplementary information [file 41598_2017_12000_MOESM1_ESM.pdf]

Performance-based ability emotional intelligence benefits working memory capacity during performance on hot tasks.

María José Gutiérrez-Cobo[1], Rosario Cabello[2], and Pablo Fernández-Berrocal[1*]

[1] Department of Basic Psychology, Faculty of Psychology, University of Málaga, Málaga, Spain

[2] Department of Developmental and Educational Psychology, University of Granada, Granada, Spain

* Correspondence: berrocal@uma.es

Supplementary Table S1. Descriptive statistics of the EI instruments

	**Mean**	**Min.**	**Max.**	**SD**	**Median**	**Asymmetry**	**Kurtosis**	***α***
**MSCEIT**								
1. Total	108.83	86.49	124.38	8.39	110.57	-.68	-.13	.82
2. Perceiving emotions	108.32	56.33	122.29	11.33	110.96	-1.71	4.32	.78
3. Facilitating emotions	101.65	71.41	119.50	9.60	102.91	-.90	0.83	.72
4. Understanding emotions	108.67	69.73	127.00	9.62	110.80	-1.24	2.00	.76
5. Managing emotions	109.44	71.91	124.20	10.71	111.64	-1.03	1.06	.78
**TMMS**								
6. Attention to feeling	3.52	1.00	5.00	.74	3.63	-.62	.34	.90
7. Clarity of feeling	3.42	1.00	5.00	.72	3.50	-.42	.40	.85
8. Mood repair	3.44	1.50	5.00	.70	3.50	-.34	.23	.85
**EQi:S**								
9. Interpersonal	4.23	2.29	5.00	.44	4.29	-.59	1.46	.78
10. Adaptability	3.80	1.20	5.00	.66	3.80	-.55	.93	.82
11. Stress management	3.35	1.13	4.88	.84	3.5	-.30	-.44	.87
12. Intrapersonal	3.72	1.88	5.00	.67	3.75	-.44	-.17	.81

Supplementary Table S2. Pearson correlations between the EI instruments

**MSCEIT**	1	2	3	4	5	6	7	8	9	10	11
1. Total											
2. Perceiving emotions	.72**										
3. Facilitating emotions	.71**	.44**									
4. Understanding emotions	.60**	.11	.23**								
5. Managing emotions	.50**	.03	.20**	.27**							
**TMMS**											
6. Attention to feeling	.00	–.09	.01	.01	.14						
7. Clarity of feeling	.00	–.12	.01	.02	.18*	.26**					
8. Mood repair	.10	.00	.10	.03	.18*	.08	.43**				
**EQi:S**											
9. Interpersonal	.09	.06	.10	–.05	.14	.45**	.22**	.34**			
10. Adaptability	.05	–.01	-.02	.07	.10	.23**	.37**	.36**	.21**		
11. Stress management	.12	.05	.11	.09	.07	-.22**	.26**	.13	-.06	.25**	
12. Intrapersonal	.10	.00	.10	.04	.16*	-.02	.59**	.34**	.24**	.30**	.38**

*p = .05. **p = .01
